# Selective associations between cognitive practice effects and reactive balance adaptation in multiple sclerosis

**DOI:** 10.1093/arclin/acag077

**Published:** 2026-09-24

**Authors:** Maria J C Hall, Lisa Stevenson, Ruth Davidson, Sinéad Macerlane, Euan Mulvihill, Fiona McIntyre, Seán Cuttle, Daniel S Peterson, Andrew S Monaghan

**Affiliations:** School of Psychology, Queen’s University Belfast, Belfast, BT9 5BN, UK; School of Psychology, Queen’s University Belfast, Belfast, BT9 5BN, UK; School of Psychology, Queen’s University Belfast, Belfast, BT9 5BN, UK; School of Psychology, Queen’s University Belfast, Belfast, BT9 5BN, UK; School of Psychology, Queen’s University Belfast, Belfast, BT9 5BN, UK; School of Psychology, Queen’s University Belfast, Belfast, BT9 5BN, UK; School of Psychology, Queen’s University Belfast, Belfast, BT9 5BN, UK; College of Health Solutions, Arizona State University, Phoenix, AZ 85004, USA; Phoenix Veterans Affairs Healthcare System, Phoenix, AZ 85012**,** USA; School of Psychology, Queen’s University Belfast, Belfast, BT9 5BN, UK

**Keywords:** multiple sclerosis, practice effects, cognition, balance training, motor learning, neurorehabilitation

## Abstract

**Objective:**

Cognitive and balance impairments are common in multiple sclerosis (MS). Practice effects may reflect neuroplasticity and predict rehabilitation responsiveness. This study examined cognitive practice effects and their relationship to reactive balance adaptation in MS.

**Methods:**

Twenty-seven people with MS and 17 neurotypical adults completed 2 baseline cognitive assessments over 2 weeks, followed by a 2-week perturbation-based balance training program over 6 progressive sessions. Verbal learning and processing speed were assessed using the California Verbal Learning Test-II (CVLT-II) and the Symbol Digit Modalities Test. Reactive balance was evaluated using backward margin of stability, step latency, and step length, pre-, post-, and 2 months after training.

**Results:**

People with MS improved on verbal learning (CVLT-II Total Recall: F (1, 42) = 83.11, *p* < .001, η^2^p = 0.66) and processing speed (F(1, 42) = 4.68, *p* = .036, η^2^p = 0.10). The magnitude of improvement did not differ significantly between groups; however, standardized regression-based scores indicated the MS group’s gains were smaller than normatively expected (t(26) = −2.60, *p* = .015, d = −0.50). Only delayed recall predicted immediate improvements in backward margin of stability (β = 0.71, *p* = .033, η^2^p = 0.22) and, with some sensitivity to influential observations, retained improvements at two-month follow-up (β = 1.12, *p* = .015, η^2^p = 0.29).

**Conclusions:**

Cognitive practice effects in MS are largely preserved. Delayed verbal recall was selectively associated with reactive balance adaptation, suggesting a possible role for consolidation-dependent memory in balance rehabilitation, though this association requires confirmatory replication.

## Introduction

Multiple sclerosis (MS) is a chronic neurodegenerative and inflammatory disease of the central nervous system, affecting over 1.8 million people worldwide (Khan & Hashim, 2025). Cognitive and balance impairments are highly prevalent in MS. Approximately 41% of people with MS (PwMS) report cognitive impairment (Askari et al., 2024), with deficits commonly observed in processing speed, attention, memory, and executive function (Nasirzadeh et al., 2024). Balance dysfunction is even more widespread, affecting over 75% of PwMS, and includes increased postural sway, reduced stability limits, and delayed automatic postural reactions (Comber et al., 2018; Peterson et al., 2016). Although cognitive impairment has been well-documented in MS, one important but underexplored phenomenon is practice effects, improvements in test performance due to repeated exposure, and their potential implications for treatment responsiveness.

Practice effects have been recognized as a clinically meaningful marker in several neurological conditions, with relevance for diagnosis, prognosis, and treatment monitoring (Duff et al., 2007; Jones, 2015). In healthy older adults, robust practice effects are typically observed on neuropsychological tests; however, in MS, findings are weaker and more variable (Holm et al., 2022). For example, on the Symbol Digit Modalities Test (SDMT), a key measure of processing speed, practice gains are modest and often approach the minimal clinically important difference (≈3–4 points), with performance plateauing after multiple assessments (Novakova et al., 2025). Similarly, on the California Verbal Learning Test-II (CVLT-II), initial small gains plateau after two administrations (Edgar et al., 2011).

Digital and smartphone-based cognitive testing has further demonstrated domain-specific effects, with strong practice effects observed in tasks requiring attention or dexterity but short-lived or absent practice effects in mobility-related measures in people with MS (Holm et al., 2022). Importantly, the absence of practice effects appears to be prognostically relevant: Individuals with low baseline processing speed and minimal practice effects are at significantly higher risk of cognitive decline over time, whereas those with preserved practice effects tend to maintain or improve their cognitive trajectories (Duff et al., 2011). In MS specifically, reduced practice effects are linked to greater lesion burden, cortical atrophy, and fatigue, factors known to impair cognitive plasticity (Jakimovski et al., 2024). Beyond MS, diminished practice effects have been proposed as an early marker of neurodegenerative risk across disorders (Jutten et al., 2020). Collectively, these findings suggest that blunted practice effects in MS may reflect reduced neural adaptability and could serve as a sensitive prognostic indicator of disease progression.

Practice effects may also influence rehabilitation outcomes. Reactive balance, the rapid, automatic stepping or grasping response to a sudden loss of stability, is impaired in PwMS, who exhibit slower protective steps, larger center-of-mass displacements, and delayed recovery, particularly during backward stepping (Liew et al., 2021; Mohamed Suhaimy et al., 2020; Peterson et al., 2016). Perturbation-based balance training (PBT), which repeatedly exposes individuals to unexpected balance disturbances, has been shown to improve step latency and margin of stability in PwMS, with gains retained for at least 2 months (Mohamed Suhaimy et al., 2020; Monaghan et al., 2023b; Peterson et al., 2025).

Systematic reviews and meta-analyses corroborate these findings, indicating that PBT substantially improves reactive balance responses but that long-term reductions in fall rates are inconsistent and vary across individuals (Sharma et al., 2025; Wallin et al., 2024). The adaptability of reactive balance in PwMS is underscored by the improvements in step latency and stability margins following even short training programs (Peterson et al., 2025). However, not all individuals benefit equally, and emerging evidence from Parkinson’s Disease and aging research suggests that interindividual variability in responsiveness may partly reflect cognitive and executive control mechanisms that influence motor adaptation (Monaghan et al., 2023a; Nørgaard et al., 2024; Pelicioni et al., 2019).

The current study aims to evaluate whether PwMS exhibit practice effects over a 2-week period, compare these effects to neurotypical controls, and determine whether practice effect magnitude predicts responsiveness to balance training. We hypothesize that practice effects will be reduced in PwMS compared to controls, and that greater practice effects will be associated with larger improvements in reactive balance.

## Methods

The dataset used in this study was previously reported in (Peterson et al., 2025), which examined the effectiveness of a 2-week reactive balance training program in PwMS. The current work extends those findings by specifically investigating cognitive practice effects and their relationship to balance performance, offering a novel contribution to the field.

### Participants

A total of 27 individuals with clinically confirmed multiple sclerosis (PwMS; mean age = 56.5 ± 14.0 years) and 17 age- and sex-matched neurotypical controls were included in the study. PwMS were recruited through physician referral, electronic medical records from the Phoenix VA Medical Center, National MS Society referrals, and local support groups across Phoenix and Flagstaff. MS diagnosis was established by the participant’s treating neurologist and confirmed via review of medical records (including, where applicable, Phoenix VA Medical Center electronic health records) prior to enrollment. Inclusion criteria for PwMS were age 18–80 years, no neurological conditions other than MS, and elevated fall risk defined by at least one fall in the previous year, an Activities-Specific Balance Confidence (ABC) Scale score below 80 (Mak & Pang, 2009), or a Dynamic Gait Index (DGI) score below 19 (Dibble & Lange, 2006). Controls met the same age and standing requirements but had no history of neurological disease or elevated fall risk. Demographic and disease-specific characteristics are presented in Table 1. Ethical approval was obtained from Arizona State University and the Phoenix VA Medical Centre Institutional Review Boards, and the study was registered on ClinicalTrials.gov as *Protective Step Training & MS* (NCT03551665). All participants provided written informed consent.

**Table 1:** Participant characteristics for people with multiple sclerosis and neurotypical controls

	NT control	PwMS		
**Variable**	**Mean (SD)**	**Mean (SD)**	**Statistic**	***p***
N (Female)	17 (11)	27 (16)		
Age	58.31 (19.30)	56.49 (13.97)	0.34	.74
Mini-BESTest (/28)	25.63 (1.93)	19.62 (5.38)	5.18	<.001
MoCA (/30)	27.25 (2.18)	26.37 (2.47)	1.22	.23
ABC (/100)	96.66 (4.92)	70.72 (11.83)	9.49	<.001
Falls in Previous Year = Yes (%)	3 (17.6)	16 (59.3)	*Z* = 2.71	.01
PDDS (/8)	—	2.69 (1.85)	—	—
MS Subtype *n* (%)				
RRMS	—	23 (85.20)	—	—
PPMS	—	3 (11.10)	—	—
SPMS	—	1 (3.70)	—	—
EDMUS (/8) *n* (%)				
2	—	3 (11.10)	—	—
3	—	3 (11.10)	—	—
4	—	7 (25.90)	—	—
5	—	6 (22.20)	—	—
6	—	5 (18.50)	—	—
6.5	—	2 (7.40)	—	—
7	—	1 (3.70)	—	—

*Note:* Values are presented as mean (SD) unless otherwise indicated. Group differences for continuous variables were assessed using independent-samples Welch t-tests; the fall history comparison used a two-proportion z-test. The statistic column reports t for continuous variables and *Z* for the fall history proportion comparison. *p* values are unadjusted and two-tailed. MiniBEST = Mini Balance Evaluation Systems Test; MoCA = Montreal Cognitive Assessment; ABC = Activities-Specific Balance Confidence Scale. MS subtypes are reported as counts (%): RRMS = relapsing–remitting multiple sclerosis; PPMS = primary progressive multiple sclerosis; SPMS = secondary progressive multiple sclerosis.

### Study design and protocol

A multiple-baseline study design spanning 18 weeks was used to evaluate changes in reactive balance and cognition. At the initial screening visit, assessments included MS-related disability using the Patient Determined Disease Steps (PDDS) (Learmonth et al., 2013) and the European Database for Multiple Sclerosis (EDMUS) (Confavreux et al., 1992), balance (MiniBESTest) (Leddy et al., 2011), and global cognition (Montreal Cognitive Assessment-MoCA) (Gill et al., 2008; Marinus et al., 2003). Reactive stepping assessments for this protocol have been discussed previously (Peterson et al., 2025). Briefly, reactive stepping and cognitive assessments occurred at baseline visits 1 and 2 (B1, B2), with a 2-week interval between them. Then, participants completed a 2-week reactive step training period. After the training, participants underwent reactive stepping assessments at the post-training assessment (P1). Finally, a retention assessment, post 2 (P2), was conducted 2 months after P1 to gauge retention.

### Cognitive assessments

Cognition was assessed at Baseline 1 (B1) and Baseline 2 (B2) using two validated neuropsychological instruments. The California Verbal Learning Test-Second Edition (CVLT-II) (Delis et al., 2016) evaluated verbal learning and memory across five immediate recall trials (Total Recall; max = 80), Short-Delay Free Recall (max = 16), and Long-Delay Free Recall (max = 16). The SDMT (Smith, 1973) measured information processing speed, with the total number of correct responses recorded in 90 s.

To maximize sensitivity to practice effects, alternate forms were not used at B2, consistent with findings that repeated administration of identical SDMT and CVLT-II forms captures learning-related change without substantial fatigue or test–retest noise (Novakova et al., 2025; van Oirschot et al., 2020). Both tests are core components of the Brief International Cognitive Assessment for MS (BICAMS), a standardized international toolset demonstrating robust reliability and validity across populations (Barcellos et al., 2021; Drulović et al., 2022; Niino et al., 2017). Test–retest reliability coefficients typically range from r = 0.80–0.93 for the SDMT and r = 0.70–0.85 for the CVLT-II, supporting their suitability for short-term reassessment in PwMS (Farghaly et al., 2021; Goretti et al., 2014).

### Reactive balance assessments

Reactive stepping was assessed at B1, B2, P1, and P2 using support-surface perturbations delivered on a dual-belt Bertec instrumented treadmill (Bertec Corp., Columbus, OH). Perturbations followed a ramped profile (300 ms acceleration, 500 ms constant speed, 300 ms deceleration) in forward and backward directions. The minimal acceleration at B1 that reliably elicited a single reactive step without inducing a fall was used across sessions. Participants wore an overhead safety harness that prevented falls but did not assist stepping; harness support was defined as >10% body weight supported.

At each session, participants completed eight warm-up perturbations (two per direction), followed by six randomized assessment trials (three forward, three backward). Kinematics were captured with a 14-camera motion analysis system (100 Hz; Motion Analysis Corporation, Santa Rosa, CA) synchronized with Bertec force plates (2,000 Hz). Marker clusters were affixed bilaterally to the feet, shank, thigh, sacrum, and T12 vertebrae. Marker trajectories were low-pass filtered at 20 Hz, and force data at 10 Hz. The primary reactive balance outcome was the anterior–posterior margin of stability (MOS) as this was the a priori primary outcome in the pre-registered clinical trial. MOS was defined as the anteroposterior displacement of the extrapolated center of mass relative to the base of support at first foot contact (Hof et al., 2005). Step latency, computed as the time from perturbation onset to step initiation, and step length, the anteroposterior distance between feet at first foot contact, were secondary reactive balance outcomes. Backward stepping outcomes were prioritized a priori, as backward balance control is disproportionately impaired in PwMS (Monaghan et al., 2022; Peterson et al., 2016).

### Perturbation-based training intervention

Following B2, PwMS completed six 45-min training sessions across 2 weeks (3 sessions/week). Each session comprised 32 perturbations (8 forward, 8 backward, 8 leftward, 8 rightward) delivered in four-trial blocks. Perturbation intensity was adjusted using an adaptive harness support paradigm: acceleration increased by 0.2 m/s^2^ if no harness catches occurred, remained unchanged if one occurred, and decreased if two or more occurred (Peterson et al., 2025). This closed-loop progression individualized challenge intensity while ensuring safety.

### Statistical analysis

Practice effects were quantified using two complementary approaches. First, absolute change scores (B2 - B1) were computed for each cognitive measure and examined using mixed ANOVAs (see below), reflecting the raw magnitude of cognitive change over the baseline period. Second, standardized regression-based (SRB) scores were computed for PwMS only, indexing each participant’s observed B2 performance relative to the score predicted by the neurotypical control regression model. These approaches answer distinct questions: absolute change scores indicate whether performance improved, whereas SRB scores indicate whether that improvement was larger or smaller than normatively expected. A participant may show a robust absolute practice effect yet still have a negative SRB score if their gain was smaller than the control-derived prediction.

First, a series of 2 × 2 mixed ANOVAs was conducted for each cognitive measure (SDMT, CVLT-II Short-Delay Free Recall, CVLT-II Long-Delay Free Recall, CVLT-II Total Recall). Group (MS vs. neurotypical controls) served as the between-subjects factor, while Time (Baseline 1 [B1] vs. Baseline 2 [B2]) was the within-subjects factor. This method addressed whether cognitive performance changed over the 2-week baseline period (main effect of Time), whether the groups differed overall (main effect of Group), and whether the change from B1 to B2 differed between groups (Group × Time interaction). Significant main effects of Time and significant Group × Time interactions were followed by Bonferroni-corrected pairwise comparisons of estimated marginal means to identify the direction of effects. Partial eta-squared (η^2^p) was used as the effect size measure.

Second, practice effects for CVLT-II Long-Delay Free Recall, Short-Delay Free Recall, and Total Recall (Trials 1–5), and the SDMT were quantified using the SRB approach, informed by previously validated methods for modeling reliable cognitive change (Duff, 2014; Hammers et al., 2021). SRB-predicted retest scores were calculated using means, standard deviations, and reliability coefficients from our age-matched neurotypical control cohort (Table 1). For each participant with MS, a z-score was computed to reflect the difference between their observed and predicted retest score, standardized by the estimate of error. Consistent with clinical convention, z-scores exceeding ±1.645 were interpreted as significant at the 90% confidence level (Duff et al., 2007). Importantly, SRB scores were calculated for PwMS only, as the neurotypical control cohort served as the normative reference sample from which the regression parameters were derived. Group-level practice effects were evaluated using one-sample t-tests comparing mean z-scores to zero, indicating systematic improvement or decline relative to our neurotypical control cohort.

For each cognitive outcome (SDMT, CVLT-II), simple change scores were entered as predictors of immediate (P1–B2) and retained (P2–B2) changes in the primary reactive balance outcome, MOS, and the secondary outcomes, step length and step latency. The association between CVLT-II Long-Delay Free Recall change and immediate (P1-B2) anteroposterior margin of stability (AP MOS) improvement was designated the primary comparison, as AP MOS was the pre-registered primary balance outcome (Peterson et al., 2025). All other associations are exploratory and hypothesis-generating with no correction for multiple comparisons. However, Benjamini–Hochberg FDR and Holm–Bonferroni corrections were subsequently applied across all 24 models to assess robustness to multiple comparisons (Benjamini & Hochberg, 1995) (Supplemental Table 3). Regression models were specified as simple linear regressions with a single cognitive predictor to preserve statistical power given the available sample sizes; covariates (e.g., baseline balance ability, age, disease severity) were, therefore, not included in the primary models. Sensitivity analyses excluded influential data points identified by Cook’s distance (>4/n) and standardized residuals (>|2|) following recommended regression diagnostic standards. For each model, we report the unstandardized regression coefficient (β), 95% confidence interval, *p*-value, Pearson correlation coefficient (r), and partial eta-squared (η^2^) to quantify effect size. All analyses were performed in R. Given the sample sizes available (*n* = 20–21 per regression model), the study had 80% power to detect effects of *r* ≥ 0.54 (η^2^p ≥ 0.29); minimum detectable effect sizes for all models are reported in Supplemental Table 2. Alpha was set at 0.05 (two-tailed), with effect sizes and 95% confidence intervals reported. This study was retrospectively registered on the Open Science Framework (https://osf.io/h4fgk).

## Results

### Participant retention and training adherence

Refer to the CONSORT diagram in Peterson and colleagues (2025) for a detailed overview of recruitment and dropout (Peterson et al., 2025). In summary, 38 individuals enrolled in the study, with 27 completing both the training and immediate post-assessment (P1). Out of these, 20 participants completed the 2-month follow-up assessment (P2). The authors report that participants, on average, completed 96.9% (SD: 9.29) of their training dose, and no adverse events were recorded.

### Reactive balance outcomes

Results of reactive balance training have been reported previously (Peterson et al., 2025). In summary, backward reactive stepping showed no exposure effects across baseline sessions (all *p* ≥ .603), but demonstrated significant immediate post-training improvements in the margin of stability (MOS; *β* = 0.029 m, 95% CI [0.002, 0.056], *p* = .036) and step latency (*β* = 0.041 s, 95% CI [0.009, 0.073], *p* = .012), with step-latency gains retained at 2-month follow-up (*β* = 0.039 s, 95% CI [0.003, 0.074], *p* = .033). MOS and step length were not retained (*p* ≥ .165).

### Cognitive practice effects: raw change scores

**Figure 1:**
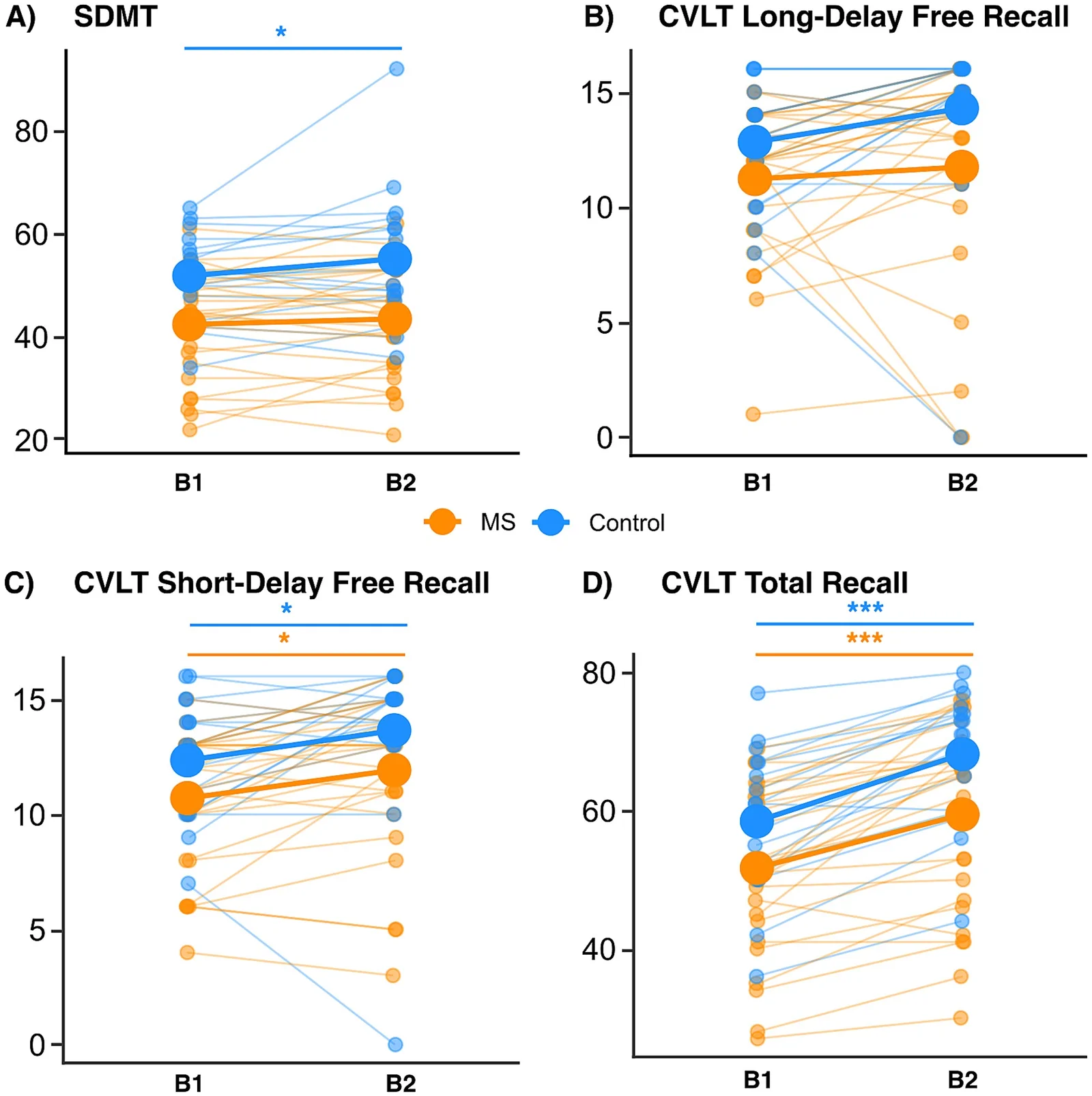
Practice effects across cognitive measures in people with multiple sclerosis (MS) and neurotypical controls from Baseline 1 (B1) to Baseline 2 (B2). Small dots represent individual participants; large dots and connecting lines represent group means. Asterisks indicate significant within-group improvements (* *p* < .05, *** *p* < .001). No significant between-group differences in the magnitude of practice effects were observed for any measure (all Group × Time interactions *p* ≥ .278). SDMT = Symbol Digit Modalities Test; CVLT-II = California Verbal Learning Test–Second Edition.

Both groups exhibited statistically significant, albeit modest improvement on the SDMT from B1 to B2 (Time: F(1,42) = 4.68, *p* = .036, η^2^p = 0.10) (Fig. 1; Table 2), and controls outperformed PwMS overall (Group: F(1, 42) = 11.20, *p* = 0.002, η^2^p = 0.21; Table 2). The Group × Time interaction was not significant (F(1, 42) = 1.21, *p* = .278, η^2^p = 0.03); follow-up simple effects indicated the Time effect was driven primarily by controls (*p* = .043) rather than PwMS (*p* = .397).

**Table 2:** Cognitive practice effects from Baseline 1 to Baseline 2 in people with multiple Sclerosis and neurotypical controls

Cognitive score	Group	B1 (SD)	B2 (SD)	Δ B2–B1 (95% CI)	Mixed ANOVA		
					**Time *F* (**_**1, 42)**_	**Group *F*** _**(1,42)**_	**G × T. *F*** _**(1,42)**_
SDMT	MS	42.44 (10.33)	43.52 (10.59)	1.07 (−1.14, 3.29)	***F*** **= 4.68 *p* = .036 *η***^***2***^***p* = 0.10**	***F* = 11.20 *p* = .002 *η*** ^***2***^ ***p* = 0.21**	*F* = 1.21, *p* = .278 *η^2^p* = 0.03
	Control	51.88 (8.44)	55.18 (13.15)	3.29 (−0.70, 7.29)			
CVLT Short-Delay Free Recall (/16)	MS	10.70 (2.99)	11.93 (3.40)	1.22 (0.49, 1.95)	***F* = 11.27 *p* = .002 *η*** ^***2***^***p* = 0.21**	*F* = 3.31 *p* = .076 *η^2^p* = 0.07	*F* = 0.01 *p* = .924 *η^2^p* = <0.01
	Control	12.35 (2.62)	13.65 (3.82)	1.29 (−0.32, 2.91)			
CVLT Long-Delay Free Recall (/16)	MS	11.22 (3.23)	11.74 (4.71)	0.52 (−1.05, 2.09)	*F* = 3.06, *p* = .088 *η^2^p* = 0.07	***F* = 4.17 *p* = .047 *η*** ^***2***^***p* = 0.09**	*F* = 0.70 *p* = .407 *η^2^p* = 0.02
	Control	12.82 (2.48)	14.29 (3.90)	1.47 (−0.15, 3.09)			
CVLT Trial 1–5 Total Recall (/80)	MS	51.70 (12.03)	59.44 (13.48)	7.74 (5.13, 10.35)	***F* = 83.11 *p* = <.001 *η*** ^***2***^ ***p* = 0.66**	***F* = 4.80 *p* = .034 *η*** ^***2***^ ***p* = 0.10**	*F* = 1.05 *p* = .310 *η^2^p* = 0.02
	Control	58.47 (10.68)	68.18 (9.53)	9.71 (6.91, 12.50)			

*Note:* Values are Mean (SD). Δ = mean change score (B2 - B1) with 95% confidence interval in brackets. The Time column reports the main effect of Time from the mixed ANOVA (Group × Time); Int. = Group × Time interaction. *η^2^p* = partial eta-squared. SDMT = Symbol Digit Modalities Test-Second Edition; CVLT-II = California Verbal Learning Test-Second Edition (CVLT-II). Bold values indicate statistically significant effects (p < .05).

Short-delay recall improved significantly in both groups from B1 to B2 (Time: F(1, 42) = 11.27, *p* = .002, η^2^p = 0.21; simple effects: PwMS *p* = .012, controls *p* = .033). Neither the Group main effect (F(1, 42) = 3.31, *p* = .076, η^2^p = 0.07) nor the Group × Time interaction (F(1, 42) = 0.01, *p* = .924, η^2^p < 0.01) was significant (Table 2).

Delayed recall did not improve significantly in either group from B1 to B2 (Time: F(1, 42) = 3.06, *p* = .088, η^2^p = 0.07; simple effects: PwMS *p* = .467, controls *p* = .106), though controls scored higher overall (Group: F(1, 42) = 4.17, *p* = .047, η^2^p = 0.09). The Group × Time interaction was not significant (F(1, 42) = 0.70, *p* = .407, η^2^p = 0.02; Table 2), indicating comparable rates of change between groups despite the absence of improvement.

Total recall showed the largest practice effect of any measure, improving significantly in both groups (Time: F(1, 42) = 83.11, *p* < .001, η^2^p = 0.66; simple effects *p* < .001 for both PwMS and controls). Controls scored higher overall (Group: F(1, 42) = 4.80, *p* = .034, η^2^p = 0.10), but the Group × Time interaction was not significant (F(1, 42) = 1.05, *p* = .310, η^2^p = 0.02; Table 2), confirming comparable improvement across groups.

### Cognitive practice effects: standardized regression-based analysis

**Figure 2:**
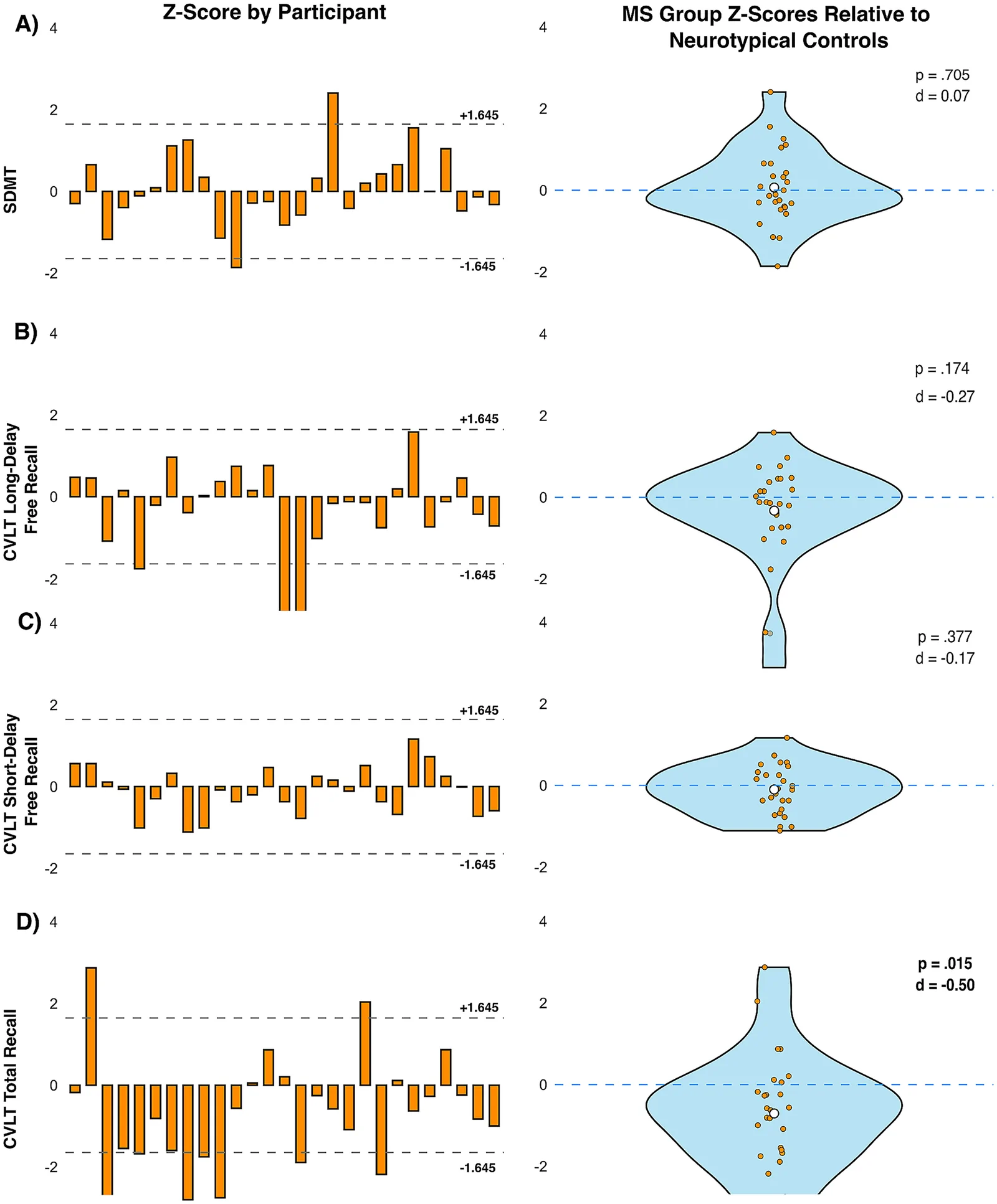
Standardized regression-based (SRB) z-scores for cognitive practice effects in people with multiple sclerosis. SRB scores are standardized to neurotypical controls (0 = expected change), with positive and negative values indicating better- or worse-than-expected performance, respectively. Dashed lines mark the ±1.645 reliable-change limits; only CVLT Total Recall showed a significant deviation from zero at the group level.

SRB scores did not differ significantly from zero for SDMT (t(26) = 0.38, *p* = .705, d = 0.07), Short-Delay Free Recall (t(26) = −0.90, *p* = .377, d = −0.17), or Long-Delay Free Recall (t(26) = −1.40, *p* = .174, d = −0.27), indicating practice effects consistent with normative expectations (Table 3). In contrast, CVLT-II Total Recall showed a significant negative SRB score (t(26) = −2.60, *p* = .015, d = −0.50; Fig. 2), the only measure for which PwMS practice effects fell short of normative prediction.

**Table 3:** Standardized regression-based (SRB) z-scores for practice effects in people with multiple sclerosis

Cognitive measure	Mean SRB *z*-score	SD	*t*	*df*	*p*	Cohen’s *d*
SDMT	0.07	0.91	0.38	26	.705	0.07
CVLT-II Short-Delay Free Recall	−0.10	0.58	−0.90	26	.377	−0.17
CVLT-II Long-Delay Free Recall	−0.33	1.21	−1.40	26	.174	−0.27
CVLT-II Total Recall	−0.71	1.41	−2.60	26	**.015**	**−0.50**

*Note:* SRB *z*-scores index B2–B1 change relative to predictions from the neurotypical control group; positive values indicate greater-than-expected improvement. SRB = standardized regression-based. Bold values indicate statistically significant effects (p < .05).

### Associations between cognitive practice effects and reactive balance adaptation

SDMT and CVLT-II Short-Delay Free Recall change scores showed minimal evidence of association with reactive stepping outcomes: neither significantly predicted immediate nor retained change in MOS, step length, or step latency in full models (all *p*’s ≥ .230, r’s ≤ 0.27, η^2^p ≤ 0.07; Supplemental Table 1). One exception emerged in the sensitivity analysis: The association between Short-Delay Free Recall change and immediate step length became significant after removing an influential observation (β = −2.05, 95% CI [−4.04, −0.06], *p* = .046, η^2^p = 0.21) and should be interpreted with caution.

**Figure 3:**
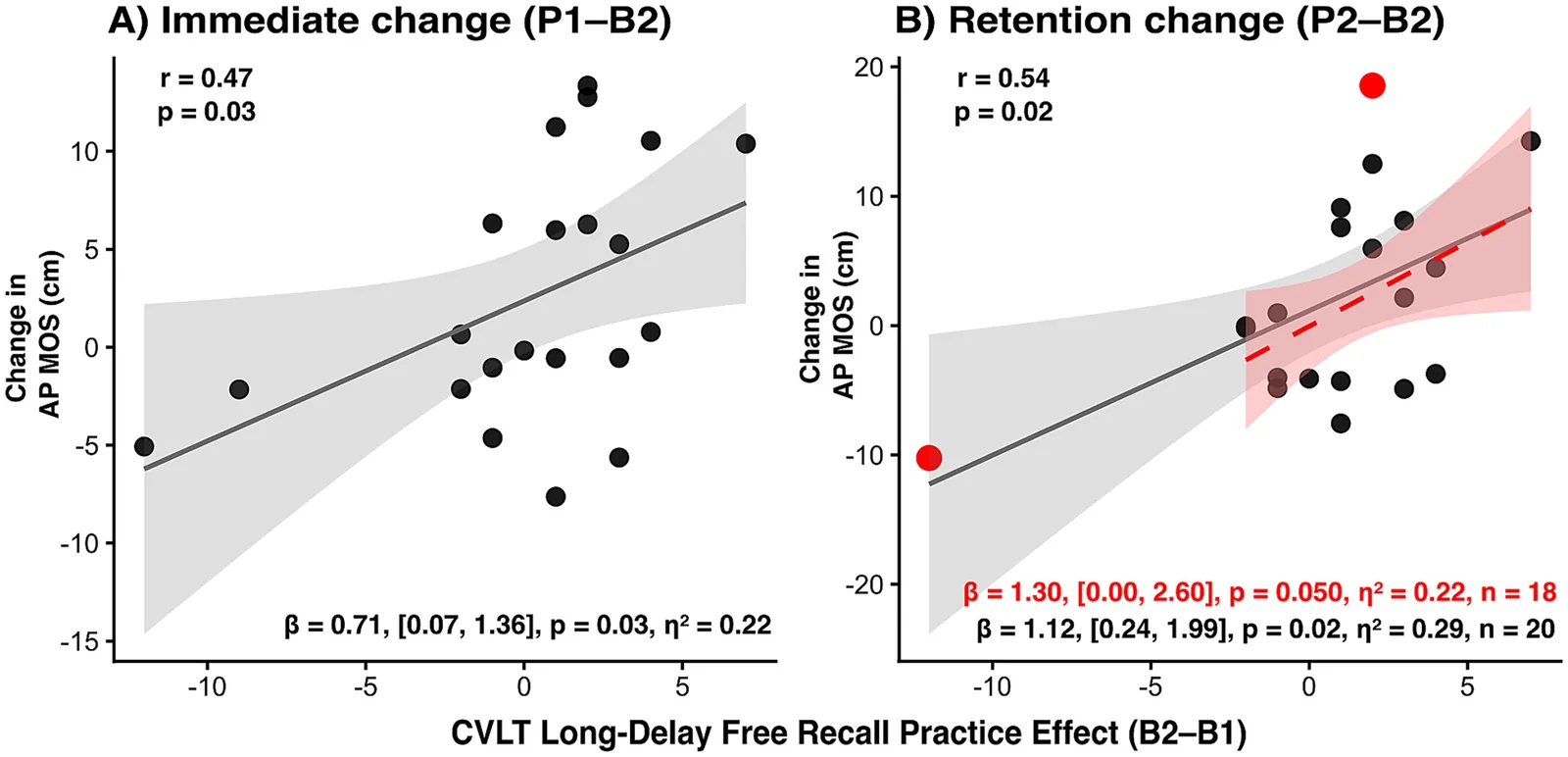
Associations between CVLT-II Long-Delay Free Recall practice effects (B2–B1) and immediate (Panel A; P1–B2) and retained (Panel B; P2–B2) improvements in anteroposterior margin of stability (AP MOS) in people with multiple sclerosis. Solid lines and shaded bands represent full-model regressions with 95% confidence intervals; statistics are displayed within each panel. No influential observations were identified in Panel A. In Panel B, two influential observations were removed in sensitivity analyses; the dashed red line represents the sensitivity model. The retained improvement association was borderline following outlier removal and should be interpreted with caution.

CVLT-II Long-Delay Free Recall change scores showed significant associations of medium-sized effect with both immediate (β = 0.71, 95% CI [0.07, 1.36], *p* = .033, *r* = 0.47, η^2^p = 0.22; no influential observations) and retained (β = 1.12, 95% CI [0.24, 1.99], *p* = .015, *r* = 0.54, η^2^p = 0.29) improvements in MOS (Fig. 3; Supplemental Table 1). The retained association was sensitive to two influential observations and dropped to borderline significance on their removal (β = 1.30, 95% CI [0.00, 2.60], *p* = .050, η^2^p = 0.22). Neither association survived Benjamini–Hochberg correction across the full family of 24 comparisons (BH-adjusted *p* = .39 and *p* = .36, respectively; Supplemental Table 3), and we interpret both as preliminary, hypothesis-generating signals. Long-Delay FR change was not associated with step length or step latency in full models (all *p*’s ≥ .116), though the retained step-length association reached significance after removing an influential observation (β = 0.85, 95% CI [0.11, 1.59], *p* = .026, η^2^p = 0.25). CVLT-II Total Recall change scores were not associated with any stepping outcome at either time point, in full or sensitivity models (all *p*’s ≥ .284, η^2^p ≤ 0.06).

## Discussion

This study investigated 2-week cognitive practice effects and their association with reactive balance adaptation in PwMS. Two main findings emerged. First, cognitive practice effects did not differ significantly between PwMS and neurotypical controls, suggesting that the magnitude of short-term cognitive gains is broadly preserved in MS. However, SRB analyses revealed that total recall gains were smaller than expected based on previously reported, standardized scores, indicating the possibility of reduced practice effects relative to controls. The selective deficit identified via SRB analysis, confined to total verbal learning, indicates that the preservation of practice effects in PwMS may not be uniform. Second, improvements in delayed verbal recall demonstrated a selective association with balance adaptation, predicting both immediate and retained improvements in backward margin of stability following perturbation-based training. These results are consistent with the possibility that consolidation-dependent memory processes play a role in motor learning in this population, though the present design cannot confirm this mechanistically.

### Cognitive practice effects in MS: normative comparisons

The mixed ANOVA results did not support our a priori hypothesis of reduced practice effects in PwMS: improvements were largely comparable between groups across all cognitive measures. Within PwMS, however, practice effects for verbal learning were moderate in magnitude, whereas processing speed showed negligible change. Notably, SRB analyses demonstrated that verbal learning gains were smaller than expected relative to previously reported, neurotypical norms, with SRB scores for total recall averaging approximately half a standard deviation below prediction. This distinction between absolute improvement and norm-referenced change underscores the utility of SRB norms to help distinguish expected performance changes from true learning gains following repeated cognitive assessments in clinical populations (Duff, 2012; Marrie et al., 2021).

Despite PwMS exhibiting lower SRB deviations in verbal learning, SRB deviations were modest for most measures, indicating that, at the group level, PwMS generally exhibited practice effects within the expected normative range. This pattern aligns with prior work suggesting that some preserved short-term gains on learning-dependent neuropsychological measures may be largely preserved, especially on memory tasks, based on systematic observations of practice effects in MS (DeLuca et al., 2004; Holm et al., 2022; Sumowski et al., 2014). It is also important to consider the relatively mild disability profile of the present sample (mean PDDS = 2.69, SD = 1.85), the majority of whom had relapsing–remitting MS (85%). Individuals with progressive disease courses and greater disease burden generally exhibit more severe cognitive impairment than those with relapsing–remitting disease (Chiaravalloti & DeLuca, 2008). The preserved practice effects observed in the present study may partly reflect the relatively well-preserved cognitive functioning of this cohort. Whether similar practice effects would generalize to samples with greater disability or progressive MS remains an important question for future research.

### Cognitive-motor associations

Change scores for processing speed and short-delay recall explained ≤7% of the variance in changes in stepping outcomes across training (*η^2^p* ≤ 0.07), with confidence intervals spanning zero, indicating limited explanatory value. In contrast, delayed verbal recall change explained approximately 22%–29% of the variance in backward MOS adaptation, representing a medium-to-large effect size. These associations were present both immediately post-training and at 2-month follow-up, with regression coefficients of similar magnitude and overlapping confidence intervals across time points. Sensitivity analyses excluding influential observations produced comparable effect size estimates, suggesting that results were not driven by isolated cases. Nonetheless, confidence intervals remained moderately wide, reflecting sample-size constraints and underscoring the exploratory nature of these findings. Neither the association between delayed recall and immediate and retained changes in MOS survived multiple comparison correction. This association is therefore best characterized as a theoretically motivated, preliminary finding, consistent in direction and magnitude across both time points, but requiring confirmatory replication in an adequately powered sample before firm conclusions can be drawn.

The specificity of associations to delayed recall, rather than processing speed, short-delay recall, or total recall, suggests that learning and retention processes may be more relevant for sustaining balance adaptations than rapid information processing. Processing speed primarily indexes the efficiency of online information processing, whereas total learning and short-delay recall are more strongly influenced by encoding and initial acquisition across repeated exposures. Delayed recall, by contrast, requires retrieval after an unfilled retention interval and is therefore more closely aligned with retention and consolidation-related memory processes that stabilize newly acquired information over time (Dayan & Cohen, 2011; Doyon et al., 2009). From this perspective, individuals who show greater gains on delayed recall may also be better able to stabilize and retain motor adaptations following balance training. This interpretation remains speculative, however, because the present study did not directly measure consolidation mechanisms.

An alternative interpretation of the present findings is that cognitive practice effects may function primarily as a marker of rehabilitation potential rather than as a mechanism directly contributing to motor learning outcomes. Individuals with MS vary substantially in neurological and cognitive reserve, disease burden, and cortical atrophy, factors that have been linked to both cognitive performance and responsiveness to rehabilitation interventions (Benedict & Zivadinov, 2011; Sumowski et al., 2009). Individuals with more preserved neural resources may be better positioned to demonstrate gains across multiple domains, resulting in both larger cognitive practice effects and greater improvement following perturbation-based balance training. The observed association between cognitive practice effects and rehabilitation outcomes may reflect a shared underlying capacity for adaptation rather than a direct causal influence of practice effects on motor learning. The present study was not designed to distinguish between these competing explanations, and future studies incorporating measures of disease burden, brain structure, and cognitive reserve will be needed to clarify the mechanisms underlying this relationship. Importantly, regardless of whether cognitive practice effects represent a causal mechanism or a prognostic marker, the present findings suggest that baseline measures of learning and retention may have utility for identifying individuals most likely to benefit from rehabilitation interventions.

The delayed recall-MOS association, while theoretically motivated, does not survive correction for multiple comparisons and should be treated as a preliminary, hypothesis-generating finding rather than a confirmed effect. Across the 24 regression models tested, only the CVLT-II Long-Delay Free Recall-MOS associations reached the conventional *p* < .05 threshold, and neither survived Benjamini–Hochberg correction for the full family. This is consistent with the power analysis, which indicated that both observed effect sizes fell at or below the minimum detectable effect for 80% power at this sample size (Supplemental Tables 2 and 3). We did not treat this as grounds to discard the finding: The hypothesis was theoretically motivated and tested against the trial’s pre-registered primary balance outcome, and the same association emerged in the predicted direction at both time points, a pattern less easily attributed to chance than a single isolated result. Replication in a larger, adequately powered, pre-registered sample is therefore needed before firm conclusions can be drawn.

### Clinical implications

Clinically, these findings suggest that measures indexing learning and retention capacity across cognitive tests may be more informative than processing speed alone when considering an individual’s potential to benefit from balance training. However, cognitive practice effects accounted for only a portion of the variance in motor adaptation, indicating that cognition is one contributor among many. Cognitive measures should therefore be integrated with biomechanical, sensory, and disease-related factors rather than used in isolation to guide rehabilitation decisions.

## Limitations and future directions

Several limitations warrant consideration. The limited sample size reduced statistical precision across all analyses. The study had 80% power to detect effects of *r* ≥ 0.54 (η^2^p ≥ 0.29), a large effect by conventional benchmarks (Cohen, 2013). Several observed associations of potential clinical interest fell below this threshold and may therefore reflect true effects that the study was underpowered to detect reliably. Minimum detectable effect sizes for all models are provided in Supplemental Table 2. We additionally note that the cognitive-motor regression analyses involved 24 separate models; applying Benjamini–Hochberg correction across this full family, neither the immediate nor retained CVLT-II Long-Delay Free Recall-MOS association survives at the conventional 0.05 threshold (Supplemental Table 3), and these findings should be treated as preliminary and hypothesis-generating pending replication in an independent, adequately powered, ideally pre-registered sample. Similarly, the sample size precluded adjustment for potentially relevant covariates in the primary regression models. Variables including age, disease severity, baseline cognitive functioning, and baseline balance performance may influence both practice effects and rehabilitation responsiveness, and their absence limits causal interpretation of the observed associations. Future adequately powered studies should incorporate these as covariates in multivariable models to clarify whether the cognitive-motor associations observed here persist after accounting for such confounds. MS diagnoses were made by participants’ treating neurologists and confirmed through medical record review, but we could not verify whether standardized diagnostic criteria were applied in each case, which limits comparability with cohorts diagnosed using explicit, criterion-based systems. Cognitive assessment focused on verbal learning and processing speed; inclusion of executive function or visuospatial learning may capture additional variance relevant to balance adaptation (Leavitt et al., 2014). Future studies with larger samples should test formal moderation models and determine whether cognitive learning indices add incremental explanatory value beyond established predictors of balance recovery.

## Conclusions

Contrary to our initial hypothesis, cognitive practice effects in PwMS were largely comparable in magnitude to those of neurotypical controls, suggesting that short-term gains on learning-dependent neuropsychological measures are broadly preserved in MS. However, SRB analyses indicated that total verbal learning gains were smaller than normatively expected, suggesting this preservation may not be uniform across all domains. These effects were nonetheless domain-specific and showed limited association with reactive stepping outcomes overall. Nevertheless, improvements in delayed verbal recall were the only cognitive measure associated with reactive stepping margin of stability. This pattern was consistent across both time points but did not survive correction for multiple comparisons and requires confirmatory replication. These findings support a nuanced view in which cognitive factors may contribute to rehabilitation outcomes and provide a theoretically grounded, empirically characterized target for future studies examining whether cognitive assessment can help identify who is most likely to benefit from perturbation-based balance rehabilitation in MS.

## Supplementary Material

Supplementary_material_acag077
