# Supplementary material for: Selective associations between cognitive practice effects and reactive balance adaptation in multiple sclerosis
Source: Arch Clin Neuropsychol. 2026 Sep 24;41(7):acag077. doi: 10.1093/arclin/acag077 (PMC13603806; doi:10.1093/arclin/acag077)
Supplement: Supplementary file 1 [file supplementary_material_acag077.zip › Supplementary_caption_acag077.docx]

**Supplementary Data**: Supplemental Table 1 and Supplemental Table 2
