# Supplementary material for: Selective associations between cognitive practice effects and reactive balance adaptation in multiple sclerosis
Source: Arch Clin Neuropsychol. 2026 Sep 24;41(7):acag077. doi: 10.1093/arclin/acag077 (PMC13603806; doi:10.1093/arclin/acag077)
Supplement: Supplementary file 1 [file supplementary_material_acag077.zip › ACNP_SupplementalMaterial_acag077.docx]

**Supplemental Table 1:** Associations Between Cognitive Practice Effects (Change Scores) and Changes in Stepping Performance Following Reactive Balance Training: Full and Sensitivity Models

| **Cognitive Variable** | **Model** | **n** | ***r*** | ***β* (95% CI)** | ***p*** | ***η²p*** |
| --- | --- | --- | --- | --- | --- | --- |
| **Immediate Change in Stepping (P1–B2)** | | | | | | |
| **AP MOS** | | | | | | |
| SDMT | Full | 21 | 0.01 | 0.01 (-0.52, 0.54) | .982 | 0.00 |
|  | Sensitivity | 18 | 0.01 | 0.30 (-0.37, 0.97) | .384 | 0.05 |
| CVLT-II Long-Delay  FR | Full | 21 | 0.47 | **0.71 (0.07, 1.36)** | **.033** | 0.22 |
|  | Sensitivity | — | — | — | — | — |
| CVLT-II Short-Delay  FR | Full | 21 | 0.00 | -0.01 (-1.68, 1.67) | .994 | 0.00 |
|  | Sensitivity | 20 | 0.00 | -0.80 (-2.77, 1.17) | .398 | 0.04 |
| CVLT-II Total  Recall | Full | 21 | -0.07 | -0.06 (-0.50, 0.37) | .757 | 0.01 |
| **Step Length** | | | | | | |
| SDMT | Full | 21 | -0.24 | -0.28 (-0.84, 0.27) | .300 | 0.06 |
|  | Sensitivity | 19 | -0.24 | -0.28 (-0.96, 0.40) | .404 | 0.04 |
| CVLT-II Long-Delay  FR | Full | 21 | 0.32 | 0.53 (-0.22, 1.29) | .156 | 0.10 |
| CVLT-II Short-Delay  FR | Full | 21 | -0.27 | -1.03 (-2.78, 0.71) | .230 | 0.07 |
|  | Sensitivity | 20 | -0.27 | -2.05 (-4.04, -0.06) | .046 | 0.21 |
| CVLT-II Total  Recall | Full | 21 | -0.25 | -0.24 (-0.69, 0.22) | .284 | 0.06 |
| **Step Latency** | | | | | | |
| SDMT | Full | 21 | -0.20 | -2.22 (-7.55, 3.11) | .394 | 0.04 |
|  | Sensitivity | 19 | -0.20 | -0.88 (-3.35, 1.59) | .476 | 0.03 |
| CVLT-II Long-Delay  FR | Full | 21 | 0.06 | 0.90 (-6.63, 8.43) | .806 | 0.00 |
|  | Sensitivity | 18 | 0.06 | 5.48 (-4.31, 15.27) | .259 | 0.08 |
| CVLT-II Short-Delay  FR | Full | 21 | -0.12 | -4.38 (-21.42, 12.66) | .597 | 0.02 |
|  | Sensitivity | 19 | -0.12 | -5.91 (-19.14, 7.32) | .383 | 0.05 |
| CVLT-II Total  Recall | Full | 21 | 0.13 | 1.19 (-3.21, 5.59) | .578 | 0.02 |
|  | Sensitivity | 20 | 0.13 | 0.64 (-2.15, 3.43) | .638 | 0.01 |
| **Retained Change in Stepping (P2–B2)** | | | | | | |
| **AP MOS** | | | | | | |
| SDMT | Full | 20 | 0.21 | 0.28 (-0.37, 0.93) | .384 | 0.04 |
|  | Sensitivity | 17 | 0.21 | 0.43 (-0.19, 1.05) | .162 | 0.13 |
| CVLT-II Long-Delay  FR | Full | 20 | 0.54 | **1.12 (0.24, 1.99)** | **.015** | 0.29 |
|  | Sensitivity | 18 | 0.54 | 1.30 (0.00, 2.60) | .050 | 0.22 |
| CVLT-II Short-Delay  FR | Full | 20 | 0.17 | 0.69 (-1.34, 2.73) | .484 | 0.03 |
|  | Sensitivity | 18 | 0.17 | -0.15 (-2.22, 1.92) | .876 | 0.00 |
| CVLT-II Total  Recall | Full | 20 | -0.06 | -0.06 (-0.59, 0.47) | .810 | 0.00 |
|  | Sensitivity | 18 | -0.06 | -0.08 (-0.68, 0.52) | .783 | 0.00 |
| **Step Length** | | | | | | |
| SDMT | Full | 20 | -0.11 | -0.20 (-1.08, 0.69) | .648 | 0.01 |
|  | Sensitivity | 18 | -0.11 | -0.29 (-0.78, 0.19) | .225 | 0.09 |
| CVLT-II Long-Delay  FR | Full | 20 | 0.36 | 1.02 (-0.28, 2.31) | .116 | 0.13 |
|  | Sensitivity | 19 | 0.36 | 0.85 (0.11, 1.59) | .026 | 0.25 |
| CVLT -IIShort-Delay  FR | Full | 20 | 0.07 | 0.41 (-2.36, 3.18) | .760 | 0.01 |
|  | Sensitivity | 18 | 0.07 | -0.61 (-2.31, 1.09) | .454 | 0.03 |
| CVLT-II Total  Recall | Full | 20 | -0.22 | -0.31 (-1.00, 0.38) | .358 | 0.05 |
|  | Sensitivity | 18 | -0.22 | -0.12 (-0.68, 0.44) | .655 | 0.01 |
| **Step Latency** | | | | | | |
| SDMT | Full | 20 | -0.27 | -2.86 (-7.97, 2.25) | .256 | 0.07 |
|  | Sensitivity | 18 | -0.27 | -1.96 (-5.33, 1.41) | .247 | 0.09 |
| CVLT-II Long-Delay  FR | Full | 20 | 0.16 | 2.70 (-5.44, 10.84) | .495 | 0.03 |
|  | Sensitivity | 18 | 0.16 | 6.64 (-3.90, 17.17) | .202 | 0.10 |
| CVLT-II Short-Delay  FR | Full | 20 | -0.08 | -2.64 (-19.08, 13.80) | .740 | 0.01 |
|  | Sensitivity | 19 | -0.08 | -1.18 (-13.36, 11.00) | .845 | 0.00 |
| CVLT-II Total  Recall | Full | 20 | 0.23 | 1.98 (-2.12, 6.09) | .324 | 0.05 |
|  | Sensitivity | 19 | 0.23 | 1.57 (-1.45, 4.59) | .295 | 0.07 |

Note**.** Each block shows a full model followed by its sensitivity model (influential observations removed via Cook’s distance > 4/n or |standardized residuals| > 2). Sensitivity rows are shown in a lighter shade; where no sensitivity row appears, no influential observations were identified. Rows shaded in yellow indicate associations where statistical significance (p < .05) differed between the full and sensitivity models. Bold values in full-model rows denote significant associations (p < .05). *β* = unstandardized regression coefficient; CI = confidence interval; *r* = Pearson correlation; *η²p* = partial eta-squared. CVLT-II = California Verbal Learning Test–Second Edition; SDMT = Symbol Digit Modalities Test.

**Supplemental Table 2.** Minimum Detectable Effect Size (MDES) for All Regression Models at 80% Power (α = .05, u = 1 Predictor)

| **Timepoint** | **Balance Outcome** | **Cognitive Predictor** | **n** | **MDES f²** | **MDES r** | **MDES η²p** |
| --- | --- | --- | --- | --- | --- | --- |
| Immediate (P1–B2) | AP MOS | SDMT | 21 | 0.415 | 0.542 | 0.293 |
|  | **AP MOS** | **CVLT Long-Delay FR** | **21** | **0.415** | **0.542** | **0.293** |
|  | AP MOS | CVLT Short-Delay FR | 21 | 0.415 | 0.542 | 0.293 |
|  | AP MOS | CVLT Total Recall | 21 | 0.415 | 0.542 | 0.293 |
|  | Step Length | SDMT | 21 | 0.415 | 0.542 | 0.293 |
|  | Step Length | CVLT Long-Delay FR | 21 | 0.415 | 0.542 | 0.293 |
|  | Step Length | CVLT Short-Delay FR | 21 | 0.415 | 0.542 | 0.293 |
|  | Step Length | CVLT Total Recall | 21 | 0.415 | 0.542 | 0.293 |
|  | Step Latency | SDMT | 21 | 0.415 | 0.542 | 0.293 |
|  | Step Latency | CVLT Long-Delay FR | 21 | 0.415 | 0.542 | 0.293 |
|  | Step Latency | CVLT Short-Delay FR | 21 | 0.415 | 0.542 | 0.293 |
|  | Step Latency | CVLT Total Recall | 21 | 0.415 | 0.542 | 0.293 |
| Retained (P2–B2) | AP MOS | SDMT | 20 | 0.439 | 0.552 | 0.305 |
|  | **AP MOS** | **CVLT Long-Delay FR** | **20** | **0.439** | **0.552** | **0.305** |
|  | AP MOS | CVLT Short-Delay FR | 20 | 0.439 | 0.552 | 0.305 |
|  | AP MOS | CVLT Total Recall | 20 | 0.439 | 0.552 | 0.305 |
|  | Step Length | SDMT | 20 | 0.439 | 0.552 | 0.305 |
|  | Step Length | CVLT Long-Delay FR | 20 | 0.439 | 0.552 | 0.305 |
|  | Step Length | CVLT Short-Delay FR | 20 | 0.439 | 0.552 | 0.305 |
|  | Step Length | CVLT Total Recall | 20 | 0.439 | 0.552 | 0.305 |
|  | Step Latency | SDMT | 20 | 0.439 | 0.552 | 0.305 |
|  | Step Latency | CVLT Long-Delay FR | 20 | 0.439 | 0.552 | 0.305 |
|  | Step Latency | CVLT Short-Delay FR | 20 | 0.439 | 0.552 | 0.305 |
|  | Step Latency | CVLT Total Recall | 20 | 0.439 | 0.552 | 0.305 |

**Note.** The table reports minimum detectable effect sizes (MDES) for all simple linear regression models examined in this study. Given the exploratory nature of the analyses and the available sample sizes, MDES are reported in preference to post-hoc observed power, which is mathematically redundant with obtained p-values and provides no additional inferential information (Hoenig & Heisey, 2001). All MDES are computed at 80% power and α = .05 for simple linear regression (one predictor, u = 1). Blue shade reflects the primary outcome.

Reference: Hoenig, J. M., & Heisey, D. M. (2001). The abuse of power: The pervasive fallacy of power calculations for data analysis. The American Statistician, 55(1), 19–24.

**Supplemental Table 3:** Benjamini-Hochberg FDR- and Holm-Corrected p-Values Across the Full Family of 24 Cognitive Practice Effect-Reactive Stepping Regression Models

| **Outcome** | **Cognitive Predictor** | **n** | **r** | **β** | **95% CI** | **Raw p** | **BH-adj. p** | **Holm-adj. p** |
| --- | --- | --- | --- | --- | --- | --- | --- | --- |
| Retained AP MOS | CVLT Long-Delay FR | 20 | .54 | 1.12 | [0.24, 1.99] | **.015** | .361 | .361 |
| Immediate AP MOS | CVLT Long-Delay FR | 21 | .47 | 0.71 | [0.07, 1.36] | **.033** | .393 | .754 |
| Retained Step Length | CVLT Long-Delay FR | 20 | .36 | 1.02 | [-0.28, 2.31] | .116 | .788 | 1.000 |
| Immediate Step Length | CVLT Long-Delay FR | 21 | .32 | 0.53 | [-0.22, 1.29] | .156 | .788 | 1.000 |
| Immediate Step Length | CVLT Short-Delay FR | 21 | -.27 | -1.03 | [-2.78, 0.71] | .230 | .788 | 1.000 |
| Retained Step Latency | SDMT | 20 | -.27 | -2.85 | [-7.96, 2.25] | .256 | .788 | 1.000 |
| Immediate Step Length | CVLT Total Recall | 21 | -.24 | -0.24 | [-0.69, 0.21] | .284 | .788 | 1.000 |
| Immediate Step Length | SDMT | 21 | -.24 | -0.28 | [-0.84, 0.27] | .300 | .788 | 1.000 |
| Retained Step Latency | CVLT Total Recall | 20 | .23 | 1.98 | [-2.12, 6.09] | .324 | .788 | 1.000 |
| Retained Step Length | CVLT Total Recall | 20 | -.22 | -0.31 | [-1.00, 0.38] | .358 | .788 | 1.000 |
| Retained AP MOS | SDMT | 20 | .21 | 0.28 | [-0.37, 0.93] | .384 | .788 | 1.000 |
| Immediate Step Latency | SDMT | 21 | -.20 | -2.22 | [-7.55, 3.11] | .394 | .788 | 1.000 |
| Retained AP MOS | CVLT Short-Delay FR | 20 | .17 | 0.69 | [-1.34, 2.73] | .484 | .848 | 1.000 |
| Retained Step Latency | CVLT Long-Delay FR | 20 | .16 | 2.70 | [-5.44, 10.84] | .495 | .848 | 1.000 |
| Immediate Step Latency | CVLT Total Recall | 21 | .13 | 1.19 | [-3.21, 5.59] | .578 | .883 | 1.000 |
| Immediate Step Latency | CVLT Short-Delay FR | 21 | -.12 | -4.38 | [-21.42, 12.66] | .597 | .883 | 1.000 |
| Retained Step Length | SDMT | 20 | -.11 | -0.20 | [-1.08, 0.69] | .648 | .883 | 1.000 |
| Retained Step Latency | CVLT Short-Delay FR | 20 | -.08 | -2.64 | [-19.08, 13.80] | .740 | .883 | 1.000 |
| Immediate AP MOS | CVLT Total Recall | 21 | -.07 | -0.07 | [-0.50, 0.37] | .757 | .883 | 1.000 |
| Retained Step Length | CVLT Short-Delay FR | 20 | .07 | 0.41 | [-2.36, 3.17] | .760 | .883 | 1.000 |
| Immediate Step Latency | CVLT Long-Delay FR | 21 | .06 | 0.90 | [-6.63, 8.43] | .806 | .883 | 1.000 |
| Retained AP MOS | CVLT Total Recall | 20 | -.06 | -0.06 | [-0.59, 0.47] | .810 | .883 | 1.000 |
| Immediate AP MOS | SDMT | 21 | .01 | 0.01 | [-0.52, 0.54] | .982 | .994 | 1.000 |
| Immediate AP MOS | CVLT Short-Delay FR | 21 | -.00 | -0.01 | [-1.68, 1.67] | .994 | .994 | 1.000 |

**Note.** All 24 simple linear regression models (4 cognitive change-score predictors x 3 balance outcomes x 2 timepoints) are treated as a single family for correction. Models are ordered by raw *p*-value. BH-adj. = Benjamini-Hochberg false discovery rate correction (Benjamini & Hochberg, 1995); Holm-adj. = Holm-Bonferroni step-down correction. No association survived either correction at the conventional .05 threshold. Bold raw *p*-values indicate *p* < .05 before correction. Yellow shading = pre-specified primary comparison (CVLT-II Long-Delay Free Recall change and immediate AP MOS improvement). β = unstandardised regression coefficient; CI = confidence interval; *r* = Pearson correlation. AP MOS = anteroposterior margin of stability; FR = Free Recall; SDMT = Symbol Digit Modalities Test.
